# Using Remotely Supervised At-Home TES for Enhancing Mental Resilience

**DOI:** 10.3389/fnhum.2022.838187

**Published:** 2022-06-09

**Authors:** Jasmina Paneva, Inge Leunissen, Teresa Schuhmann, Tom A. de Graaf, Morten Gørtz Jønsson, Balder Onarheim, Alexander T. Sack

**Affiliations:** ^1^Section Brain Stimulation and Cognition, Department of Cognitive Neuroscience, Faculty of Psychology and Neuroscience, Maastricht University, Maastricht, Netherlands; ^2^Maastricht Brain Imaging Centre (MBIC), Maastricht, Netherlands; ^3^Centre for Integrative Neuroscience (CIN), Maastricht University, Maastricht, Netherlands; ^4^PlatoScience Neurostimulation ApS, Copenhagen, Denmark; ^5^Department of Psychiatry and Neuropsychology, School for Mental Health and Neuroscience (MHeNs), Brain + Nerve Centre, Maastricht University Medical Centre+ (MUMC+), Maastricht, Netherlands

**Keywords:** noninvasive brain stimulation (NIBS), transcranial electrical stimulation (TES), transcranial direct current stimulation (tDCS), major depressive disorder (MDD), relapse prevention, mental resilience, at-home TES

## Abstract

We are in the midst of a mental health crisis with major depressive disorder being the most prevalent among mental health disorders and up to 30% of patients not responding to first-line treatments. Noninvasive Brain Stimulation (NIBS) techniques have proven to be effective in treating depression. However, there is a fundamental problem of scale. Currently, any type of NIBS treatment requires patients to repeatedly visit a clinic to receive brain stimulation by trained personnel. This is an often-insurmountable barrier to both patients and healthcare providers in terms of time and cost. In this perspective, we assess to what extent Transcranial Electrical Stimulation (TES) might be administered with remote supervision in order to address this scaling problem and enable neuroenhancement of mental resilience at home. Social, ethical, and technical challenges relating to hardware- and software-based solutions are discussed alongside the risks of stimulation under- or over-use. Solutions to provide users with a safe and transparent ongoing assessment of aptitude, tolerability, compliance, and/or misuse are proposed, including standardized training, eligibility screening, as well as compliance and side effects monitoring. Looking into the future, such neuroenhancement could be linked to prevention systems which combine home-use TES with digital sensor and mental monitoring technology to index decline in mental wellbeing and avoid relapse. Despite the described social, ethical legal, and technical challenges, the combination of remotely supervised, at-home TES setups with dedicated artificial intelligence systems could be a powerful weapon to combat the mental health crisis by bringing personalized medicine into people’s homes.

## Introduction

Mental health in European countries is a cause of growing concern. In 2018 it was estimated that mental health problems affect approximately 84 million people across the EU (OECD/European Union, 2018). Mental health problems constitute the fastest-growing family of pathologies in terms of morbidity, mortality, and socio-economic costs, which are estimated at more than 4% of GDP (>600 billion euros). The ongoing COVID-19 pandemic has exacerbated the mental health crisis further (Ćosić et al., 2020; Liu et al., 2020; Talevi et al., 2020), emphasizing the need for continued exploration and development of mental healthcare innovations.

## Major Depressive Disorder, and Non-Invasive Brain Stimulation Approaches

Among mental health disorders, Major Depressive Disorder (MDD) is the most prevalent. The prevalence of MDD has been reported as anywhere between 4.4% and 5.0% globally (Ferrari et al., 2013). Therefore, we here use MDD as an example case to assess the opportunities and challenges of remote medicine using brain stimulation approaches. The most commonly prescribed treatment options for MDD are psychotherapy and pharmacotherapy. However, not all patients respond equally well to these first-line treatments. Up to 30% of MDD patients fail to respond to at least two different antidepressant medications.

A promising new treatment approach for MDD involves Non-Invasive Brain Stimulation (NIBS), which can be used to up- or downregulate brain activity in targeted regions. NIBS includes, among other techniques, transcranial magnetic stimulation (TMS) and Transcranial Electrical Stimulation (TES). TMS was explored as an MDD treatment first, specifically to offer an alternative for treatment-resistant patients. In TMS, a burst of electric current is discharged through a coil, producing a rapidly changing magnetic field that penetrates the skull. This magnetic pulse in turn induces an electric field in the brain, depolarizing neurons to modulate both local and network activity in the cortex (Reithler et al., 2011). In recent years, based on several randomized clinical trials confirming its efficacy, TMS over the frontal cortex has been acknowledged as an effective therapy of treatment-resistant depression. Up to 50% of treatment-resistant depression patients benefit from high-frequency repetitive TMS (rTMS) to Left Dorsolateral Prefrontal Cortex (DLPFC), with a remission rate of 30% (Perera et al., 2016; Blumberger et al., 2018). The use of rTMS as a therapy for treatment resistant depression (TRD) has received CE and Food and Drug Administration (FDA) approval and is increasingly widely adopted in clinical practice (McClintock et al., 2018). Relapse rates after TMS treatment are reported to be about 10% at 6 months post-treatment (Janicak et al., 2010). Unfortunately, TMS treatment is fairly involved, generally consisting of daily visits for 4–6 weeks to a clinic where medical professionals perform the procedure. On the other hand, the side effects are minimal, and it is principally safe to use. Its greatest safety concern is seizure induction, but this is highly uncommon and has no lasting adverse effects (Lefaucheur, 2019). Other side effects reported in more than 5% of the population are discomfort at the stimulation site and headaches (Zis et al., 2020).

A second non-invasive brain stimulation technique is (low-intensity) Transcranial Electrical Stimulation (TES). TES involves the application of (minimally) two electrodes, at least one of which is placed over a brain region of interest. Low-intensity electrical current (generally around 1–2 milliAmperes) flows between the anodal and cathodal electrodes, inducing changes in cortical excitability and plasticity both locally and in connected areas (Palm et al., 2016). If the polarity of each electrode remains constant, this is referred to as Transcranial Direct Current Stimulation (tDCS). In recent years, expert panels have determined that frontal tDCS was probably (Lefaucheur et al., 2020) or definitely (Fregni et al., 2021) effective in treating depression, sometimes even with effect sizes comparable to drugs and TMS (Brunoni et al., 2016). TES has a very mild side effect profile, including most commonly tingling or itching skin sensations, erythema under the electrodes, and headaches (Brunoni et al., 2011a). With more than 30,000 registered sessions in 2016, no serious adverse events had ever been reported (Bikson et al., 2016).

## Barrier of Accessibility

The increasing acknowledgment and adoption of NIBS as a valid depression treatment approach is interesting in light of growing mental health concerns. The emergence of NIBS clinics makes this treatment accessible to more and more patients, and many countries have now integrated NIBS treatment into their health and insurance systems. Furthermore, NIBS compares favorably with other treatment options like pharmacotherapy in several ways, including side effects. Side effects of antidepressants, such as weight gain, nausea, sexual dysfunction, and somnolence can significantly reduce patient quality of life and therefore compliance with treatment (Brunoni et al., 2012; Sauvaget et al., 2018). NIBS is noninvasive, and painless and it does not interact with other medications, making it particularly useful for patient subpopulations like the elderly (who often use multiple drugs concurrently), as well as pregnant and breastfeeding women (Brunoni et al., 2011b).

Currently, NIBS appears to offer unique advantages but cannot easily be applied at scale (as, e.g., pharmacotherapy), since patients repeatedly need to visit a clinic to be treated by trained personnel. Therefore, it is crucial to evaluate further technical opportunities that may eliminate some of these limitations, such as non-invasive brain stimulation with at-home treatment devices. At the moment, TES, as compared to TMS, seems to be the more reasonable option to explore, since TES technology is cheaper, more portable, easier to use, and has a higher tolerability and lower drop-out rate (Priori et al., 2009; Vanneste et al., 2010; Charvet et al., 2015). In the remainder of this review, we assess to what extent TES might be administered at home, with hand-held devices, after instruction, and with remote supervision.

## At-Home TES

Conceptually, both acute treatment of depression and relapse prevention could be done with TES systems. In practice, in the pursuit of such development, it would seem crucial to ensure remote supervision by a medical specialist or therapist who has full access to all data across all sessions, including patient compliance, clinical effects (monitored digitally using experience sampling methods and digital standard depression scales), general mental health monitoring (including changes in cognition, mobility performances, social isolation), and overall treatment progress. There are at least two published instances showcasing the efficacy and feasibility of home-delivered, supervised tDCS in MDD patients (Alonzo et al., 2019; Aparicio et al., 2019). Alonzo et al. (2019) assessed mood improvements, compliance, and feasibility of an 8-week, at-home tDCS administration protocol to treat MDD. They found significant mood improvements (in Montgomery–Asberg Depression Rating Scale scores), comparable with findings from clinic-based trials. Protocol adherence was excellent (6% dropout rate), and side effects were found to be minimal. Aparicio et al. (2019) followed both nontreatment-resistant and treatment-resistant patients undergoing at-home tDCS continuation therapy for relapse prevention over a 6-month period. Treatment was found to be well-tolerated and completion rates were 73.5%. Relapse rates were significantly lowered compared with a previous follow-up study (26.5% vs. 53%), especially for nontreatment-resistant patients. This preliminary evidence is promising for potential wider adoption of such at-home tDCS systems for acute treatment and possibly relapse prevention in MDD.

## Risks and Challenges of At-Home TES

### Over- and Under-Use

A safety concern with at-home TES systems is the over- or under-use in terms of frequency or intensity of stimulation. A study investigating the demographic characteristics and motivations of people who used direct-to-consumer, at-home tDCS devices, found that over 40% of participants self-administered an excessive number of sessions compared to prescribed scientific or clinical protocols, with 8.4% administering over 100 sessions on themselves (Wexler, 2018). A significant proportion of the participants (31.5%) reported they had bought and used at-home tDCS specifically to self-treat depression. Therefore, over-use of tDCS in people seeking depression (or other mental health) treatment should be considered. In the same study, some of the participants reported purposefully attempting to use stimulation intensities above the recommended 2 mA, even though their devices did not allow for such dosage. The current upper limit in terms of research into long-term effects of tDCS use is the study by Im et al. (2019). In this study, the effects of at-home tDCS use over DLPFC in patients with early Alzheimer’s disease (AD) were explored. Authors found tDCS to be safe and effective in treating both behavioral and metabolic correlates of AD over a 6-month period. The mechanisms underlying the therapeutic effects of tDCS are still not fully understood, and there is no data at present verifying the morphological or behavioral changes resulting from exposure to tDCS for longer than 6 months. While this is a risk for any use of tDCS, at-home tDCS opens the door to potentially much longer treatment paradigms, making it a more pressing issue. This lack of knowledge about long-term side effects presents one barrier to the adoption of at-home tDCS systems.

Another possible source of error associated with at-home tDCS use is the high degree of variability which stems from its montage. Small changes in either the physical placement of electrodes or in the intensity of the current passed through them can result in large differences in its effects. Electrode placement location determines the areas of greatest current density in the brain, and therefore the behavioral or clinical outcomes of stimulation (Bikson et al., 2010). Studies monitoring the physiological consequences of changing electrode placement (Nitsche and Paulus, 2000; Woods et al., 2015), as well as computational modeling of placement changes as small as 1 cm (Minhas et al., 2012; Kessler et al., 2013; Woods et al., 2015) have demonstrated how such small changes result in significant differences in the predicted or measured current flow in the brain. It is important, therefore, to address these methodological concerns. This can be reliably done through standardized hardware implementations concerning montage and impedance control, as discussed below.

## How to Do It Right: At-Home TES Systems

### Compliance Control

**Figure 1:**
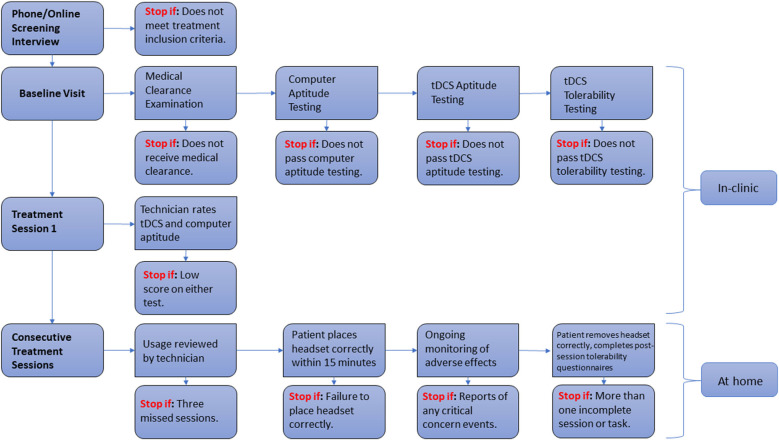
Flowchart of patient interactions with “stop” eligibility criteria. Adapted from Charvet et al. (2015).

**Figure 2:**
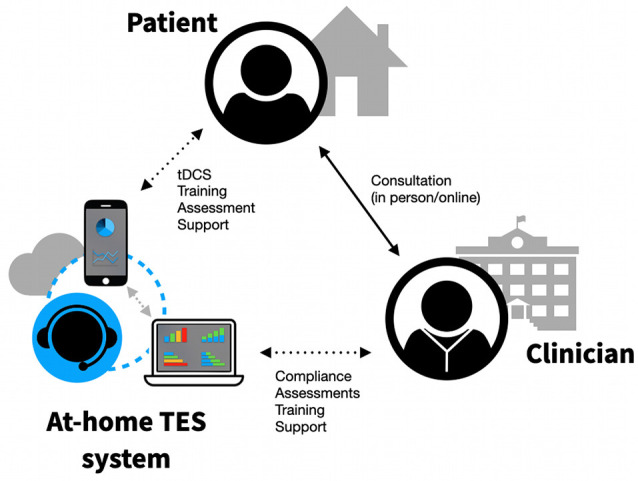
Example of a remotely supervised, at-home TES system.

The previously mentioned safety concerns might be mitigated with at-home, supervised tDCS systems for clinical trials or the provision of telemedicine. There are several key requirements which should be met in order to ensure safety, reproducibility, and consistency. First, both researchers/clinicians and patients/caregivers must be trained in tDCS administration at a rigorous and predefined standard. Training should involve formal knowledge acquisition, hands-on practical training, and supervised administration sessions. For instance, Charvet et al. (2015) proposed a decision-tree standardized flowchart of patient interactions, evaluating eligibility using “stop” criteria at each consecutive step of the process. An example of such a flowchart can be seen in Figure 1. Alongside the device itself and its accessories, the flowchart should allow researchers, technicians, and clinical staff to provide users with a safe and transparent ongoing assessment of aptitude, tolerability, compliance, and/or misuse. There is the possibility to further tailor this approach to each patient’s specific needs—some patients might only need supervision during a pre-defined trial period, while others may need it indefinitely. Monitoring could be done through the use of secure video connections, ensuring visual confirmation of device preparation, electrode placement, impedance control, and environmental factors. Stimulation sessions would only begin following the confirmation through a video that all requirements have been met. Finally, self-reports allowing subjects and technicians to keep track of any adverse events, tolerance issues, or study-specific measures must be provided. An example system can be seen in Figure 2.

### Montage and Impedance Control

Strict dose preparation and montage are essential for proper and reproducible tDCS (Peterchev et al., 2012). With remotely supervised tDCS systems this might be achieved away from research and medical centers. Specially designed headgear may allow for consistent and correct placement on the head. Headsets can be produced in several head-sizes and come with frame-of-reference markers to align to relevant landmarks. Devices should include impedance meters, which prevent the use of the system unless all electrodes have been set up accurately. All components must be clearly labeled to prevent confusion and errors in the application at home. To reduce contamination, sponges should never be reused between patients. Dose control can be set remotely, using either hardware or software solutions. Hardware solutions include pre-programmable devices, set to only provide a certain number of sessions with pre-defined parameters. The downside of such hardware-based solutions is the necessity to reconfigure/reset devices after the limited number of sessions has been exhausted. Software-based solutions are centered around devices which use the pre-generated device unlock codes. Device unlock codes can contain information about any and all relevant parameters—stimulation intensity, duration, and condition, as well as the number of sessions to be executed. Unlock codes would be provided per session by the research or medical staff, allowing for real-time feedback and control of each session. Unlock codes are not to be programmed by the researchers or caregivers, making them well-suited to the execution of double-blind clinical trials, as well as enhancing reproducibility and safety.

## Legal Status

Any device meant for research or medical use needs to comply with ethical and safety standards, for example, set by the Food and Drug Administration (FDA) in the USA or the Medical Device Directive (MDD) and health authorities in the EU. Any non-invasive brain stimulation devices used in the EU are classified as Class IIa, according to the Council Directive 93/42/CEE for medical devices, and should conform to standards and directives (Antal et al., 2019). All devices intended for clinical use must be CE marked. This means they must fulfill essential requirements for safety and performance. It is required to have systematic procedures in place in order to track the appearance and frequency of side effects and/or malfunctions by the device. Additionally, medical practitioners are required by law to report any and all incidents related to the device. Regarding cyber security, industry-standard measures should be applied, and devices should comply with safety requirements as stipulated in the new EU regulations MDR 2017/745. It is further recommended that there are two additional layers of safety applied in such devices. First, concerning hardware limitations, the inclusion of component and dedicated circuits, battery capacity, and type, in the electronics so that—regardless of software commands—it is physically impossible for the device to deliver currents above the recommended thresholds. Second, in relation to firmware limitations, the firmware installed directly on the electronics should have a separate security level, ensuring that only pre-defined and approved software messages will be executed by the firmware, and that no instructions with potentially harmful effects will be attempted or executed. With both these in place, even in scenarios where patients seek to use illicitly obtained “unlocks”, or malicious actors seek to remotely interfere with stimulation protocols, the outcome of such security breaches would still not be of any risk to the user. Finally, for any device to be approved for clinical use, there needs to be sufficient RCT evidence of therapeutic effects.

## Future Perspectives

One approach to deal with the rising demand for mental health care has been telemedicine. Telemedicine can be broadly described as any medical activity which involves an element of distance (Wootton, 2001). When it pertains to mental health, “e-mental health” is used in place of telemedicine as an umbrella term, referring to the use of any digital technologies or media, particularly those that utilize the internet, with the aim of providing acute and/or relapse prevention mental health care, across geographical distances (Riper et al., 2010). The use of e-mental health has risen sharply during the COVID-19 pandemic, due to its adaptability to social distancing and stay-at-home guidelines (Ellis et al., 2021). Its effectiveness has been shown in the context of depression (García-Lizana and Muñoz-Mayorga, 2010), anxiety (Rees and Maclaine, 2015), and post-traumatic stress disorder (Turgoose et al., 2018). At home TES solutions seem a natural fit in this larger context of telemedicine and e-health, especially if associated software innovations can learn from parallel developments in this space.

As people increasingly incorporate wearable technology to monitor vital signs, physical activity, sleep quality, and other health information, a future avenue worth exploring is the combination of such technology with telemedicine. The use of mental health apps designed for self-management, symptom tracking, cognition improvement, and social support has been on the rise with the ubiquity of mobile phone usage and are currently also increasingly prescribed by mental health practitioners as a supplementary form of care (Chandrashekar, 2018). There is evidence now suggesting that physiologic markers such as heart rate variability (HRV) can be reliable trait markers of MDD (Brunoni et al., 2013). Therefore, it has become possible to combine mobile devices capable of measuring HRV with digital platforms for the monitoring of subjective mental wellbeing, social isolation, mobility performance, and other cognitive self-report measures. This data stream can be recorded and interpreted by dedicated artificial intelligence (AI) systems, with the aim of signaling a potential transition from a healthy to a depressed mental state. Such a virtual mental health service, combining home-use tDCS treatment with a digital sensor and mental monitoring technology, could be used to index a potential decline in mental wellbeing and signal a future relapse episode to the user and their healthcare team (Gillan and Rutledge, 2021; Kelley and Gillan, 2022), indicating TES-based intervention at the earliest possible time point. Looking further ahead, there is room for optimization in the personalizing of stimulation parameters to make treatment more effective. Currently, NIBS treatments follow a one-size-fits all approach, not usually taking into account inter-individual differences in anatomy and neural dynamics (though see Evans et al., 2020 for interesting developments).

## Conclusions

The use of remotely supervised, AI-based, at-home tDCS systems for acute treatment, relapse prevention, and mental wellbeing monitoring is an avenue with innovation potential for both research and clinical practice. Scientific, societal, clinical, and financial benefits can be achieved by further development and study of such technologies. TES has been demonstrated to be easy to use, well-tolerated, efficacious, and consumer devices are already on the market. Further research into its opportunities, but also challenges, seems timely and warranted.

## Data Availability Statement

The original contributions presented in the study are included in the article, further inquiries can be directed to the corresponding author.

## Author Contributions

AS and IL conceived of the idea. JP and MJ performed the literature review. JP took the lead in writing the manuscript. All authors provided critical feedback and revisions and helped shape the manuscript. All authors contributed to the article and approved the submitted version.

## Conflict of Interest

BO is the Founder and CEO of PlatoScience ApS, Copenhagen, Denmark. MJ is the Research Manager at PlatoScience ApS. AS is scientific advisor for PlatoScience ApS. The remaining authors declare that the research was conducted in the absence of any commercial or financial relationships that could be construed as a potential conflict of interest.

## Publisher’s Note

All claims expressed in this article are solely those of the authors and do not necessarily represent those of their affiliated organizations, or those of the publisher, the editors and the reviewers. Any product that may be evaluated in this article, or claim that may be made by its manufacturer, is not guaranteed or endorsed by the publisher.

## References

[B1] Ćosić K. Popović S. Šarlija M., and Kesedžić, I. (2020). Impact of human disasters and covid-19 pandemic on mental health: potential of digital psychiatry. Psychiatr. Danub. 32, 25–31. 10.24869/psyd.2020.25 32303026

[B2] Alonzo A. Fong J. Ball N. Martin D. Chand N. Loo C. (2019). Pilot trial of home-administered transcranial direct current stimulation for the treatment of depression. J. Affect. Disord. 252, 475–483. 10.1016/j.jad.2019.04.041 31005790

[B3] Antal A. Woods A. J. Knotkova H. (2019). “Transcranial direct current stimulation ethics and professional conduct,” in Practical Guide to Transcranial Direct Current Stimulation, eds Knotkova H. Nitsche M. A. Bikson M. Woods A. J. (Cham: Springer International Publishing), 407–427. 10.1007/978-3-319-95948-1_14.

[B4] Aparicio L. V. Rosa V. Razza L. M. Sampaio-Junior B. Borrione L. Valiengo L.. (2019). Transcranial direct current stimulation (tDCS) for preventing major depressive disorder relapse: results of a 6-month follow-up. Depress. Anxiety 36, 262–268. 10.1002/da.22878 30637889

[B5] Bikson M. Datta A. Rahman A. Scaturro J. (2010). Electrode montages for tDCS and weak transcranial electrical stimulation: role of “return” electrode’s position and size. Clin. Neurophysiol. 121:1976. 10.1016/j.clinph.2010.05.020 21035740 PMC2983105

[B6] Bikson M. Grossman P. Thomas C. Zannou A. L. Jiang J. Adnan T.. (2016). Safety of transcranial direct current stimulation: evidence based update 2016. Brain Stimul. 9, 641–661. 10.1016/j.brs.2016.06.004 27372845 PMC5007190

[B7] Blumberger D. M. Vila-Rodriguez F. Thorpe K. E. Feffer K. Noda Y. Giacobbe P.. (2018). Effectiveness of theta burst versus high-frequency repetitive transcranial magnetic stimulation in patients with depression (THREE-D): a randomised non-inferiority trial. Lancet 391, 1683–1692. 10.1016/S0140-6736(18)30295-2 29726344

[B8] Brunoni A. R. Amadera J. Berbel B. Volz M. S. Rizzerio B. G. Fregni F. (2011a). A systematic review on reporting and assessment of adverse effects associated with transcranial direct current stimulation. Int. J. Neuropsychopharmacol. 14, 1133–1145. 10.1017/S1461145710001690 21320389

[B12] Brunoni A. R. Valim C. Fregni F. (2011b). Combination of noninvasive brain stimulation with pharmacotherapy. Exp. Rev. Med. Devices 8, 31–39. 10.1586/erd.10.62 21158538

[B9] Brunoni A. R. Ferrucci R. Fregni F. Boggio P. S. Priori A. (2012). Transcranial direct current stimulation for the treatment of major depressive disorder: a summary of preclinical, clinical and translational findings. Prog. Neuropsychopharmacol. Biol. Psychiatry 39, 9–16. 10.1016/j.pnpbp.2012.05.016 22651961

[B10] Brunoni A. R. Kemp A. H. Dantas E. M. Goulart A. C. Nunes M. A. Boggio P. S.. (2013). Heart rate variability is a trait marker of major depressive disorder: evidence from the sertraline vs. electric current therapy to treat depression clinical study. Int. J. Neuropsychopharmacol. 16, 1937–1949. 10.1017/S1461145713000497 23759172

[B11] Brunoni A. R. Moffa A. H. Fregni F. Palm U. Padberg F. Blumberger D. M.. (2016). Transcranial direct current stimulation for acute major depressive episodes: meta-analysis of individual patient data. Br. J. Psychiatry 208, 522–531. 10.1192/bjp.bp.115.164715 27056623 PMC4887722

[B13] Chandrashekar P. (2018). Do mental health mobile apps work: evidence and recommendations for designing high-efficacy mental health mobile apps. Mhealth 4:6. 10.21037/mhealth.2018.03.02 29682510 PMC5897664

[B14] Charvet L. E. Kasschau M. Datta A. Knotkova H. Stevens M. C. Alonzo A.. (2015). Remotely-supervised transcranial direct current stimulation (tDCS) for clinical trials: guidelines for technology and protocols. Front. Syst. Neurosci. 9:26. 10.3389/fnsys.2015.00026 25852494 PMC4362220

[B15] Ellis L. A. Meulenbroeks I. Churruca K. Pomare C. Hatem S. Harrison R.. (2021). The application of e-mental health in response to COVID-19: a scoping review and bibliometric analysis. JMIR Men. Health 8:e32948. 10.2196/32948 34666306 PMC8651237

[B16] Evans C. Bachmann C. Lee J. S. Gregoriou E. Ward N. Bestmann S. (2020). Dose-controlled tDCS reduces electric field intensity variability at a cortical target site. Brain Stimul. 13, 125–136. 10.1016/j.brs.2019.10.004 31653475

[B17] Ferrari A. J. Somerville A. J. Baxter A. J. Norman R. Patten S. B. Vos T.. (2013). Global variation in the prevalence and incidence of major depressive disorder: a systematic review of the epidemiological literature. Psychol. Med. 43, 471–481. 10.1017/S0033291712001511 22831756

[B18] Fregni F. El-Hagrassy M. M. Pacheco-Barrios K. Carvalho S. Leite J. Simis M.. (2021). Evidence-based guidelines and secondary meta-analysis for the use of transcranial direct current stimulation in neurological and psychiatric disorders. Int. J. Neuropsychopharmacol. 24, 256–313. 10.1093/ijnp/pyaa051 32710772 PMC8059493

[B19] García-Lizana F. Muñoz-Mayorga I. (2010). Telemedicine for depression: a systematic review. Perspectiv. Psychiatr. Care 46, 119–126. 10.1111/j.1744-6163.2010.00247.x 20377799

[B20] Gillan C. M. Rutledge R. B. (2021). Smartphones and the neuroscience of mental health. Annu. Rev. Neurosci. 44, 129–151. 10.1146/annurev-neuro-101220-014053 33556250 PMC9107341

[B21] Im J. J. Jeong H. Bikson M. Woods A. J. Unal G. Oh J. K.. (2019). Effects of 6-month at-home transcranial direct current stimulation on cognition and cerebral glucose metabolism in Alzheimer’s disease. Brain Stimul. 12, 1222–1228. 10.1016/j.brs.2019.06.003 31196835 PMC6703942

[B22] Janicak P. G. Nahas Z. Lisanby S. H. Solvason H. B. Sampson S. M. McDonald W. M.. (2010). Durability of clinical benefit with transcranial magnetic stimulation (TMS) in the treatment of pharmacoresistant major depression: assessment of relapse during a 6-month, multisite, open-label study. Brain Stimul. 3, 187–199. 10.1016/j.brs.2010.07.003 20965447

[B50] Kelley S. W. Gillan C. M. (2022). Using language in social media posts to study the network dynamics of depression longitudinally. Nature Commun. 13:870. 10.1038/s41467-022-28513-3. 35169166 PMC8847554

[B24] Kessler S. K. Minhas P. Woods A. J. Rosen A. Gorman C. Bikson M. (2013). Dosage considerations for transcranial direct current stimulation in children: a computational modeling study. PLoS One 8:e76112. 10.1371/journal.pone.0076112 24086698 PMC3785412

[B25] Lefaucheur J. P. (2019). Transcranial magnetic stimulation. Handbook Clin. Neurol. 160, 559–580. 10.1016/B978-0-444-64032-1.00037-0 31277876

[B26] Lefaucheur J. P. Aleman A. Baeken C. Benninger D. H. Brunelin J. Di Lazzaro V.. (2020). Evidence-based guidelines on the therapeutic use of repetitive transcranial magnetic stimulation (rTMS): an update (2014–2018). Clin. Neurophysiol. 131, 474–528. 10.1016/j.clinph.2019.11.002 31901449

[B27] Liu J. J. Bao Y. Huang X. Shi J. Lu L. (2020). Mental health considerations for children quarantined because of COVID-19. Lancet Child Adolesc. Health 4, 347–349. 10.1016/S2352-4642(20)30096-1 32224303 PMC7118598

[B28] McClintock S. M. Reti I. M. Carpenter L. L. McDonald W. M. Dubin M. Taylor S. F.. (2018). Consensus recommendations for the clinical application of repetitive transcranial magnetic stimulation (rTMS) in the treatment of depression. J. Clin. Psychiatry 79:16cs10905. 10.4088/JCP.16cs10905 28541649 PMC5846193

[B29] Minhas P. Bikson M. Woods A. J. Rosen A. R. Kessler S. K. (2012). “Transcranial direct current stimulation in pediatric brain: a computational modeling study,” in 2012 Annual International Conference of the IEEE Engineering in Medicine and Biology Society (San Diego, CA), 859–862. 10.1109/EMBC.2012.6346067 PMC3641645 23366028

[B30] Nitsche M. A. Paulus W. (2000). Excitability changes induced in the human motor cortex by weak transcranial direct current stimulation. J. Physiol. 527, 633–639. 10.1111/j.1469-7793.2000.t01-1-00633.x 10990547 PMC2270099

[B31] OECD/European Union (2018). Health at a Glance: Europe 2018: State of Health in the EU Cycle. Paris/European Union, Brussels: OECD Publishing. 10.1787/health_glance_eur-2018-en

[B32] Palm U. Hasan A. Strube W. Padberg F. (2016). tDCS for the treatment of depression: a comprehensive review. Eur. Arch. Psychiatry Clin. Neurosci. 266, 681–694. 10.1007/s00406-016-0674-9 26842422

[B33] Perera T. Yohanandan S. A. Thevathasan W. Jones M. Peppard R. Evans A. H.. (2016). Clinical validation of a precision electromagnetic tremor measurement system in participants receiving deep brain stimulation for essential tremor. Physiol. Meas. 37:1516. 10.1088/0967-3334/37/9/1516 27511464

[B34] Peterchev A. V. Wagner T. A. Miranda P. C. Nitsche M. A. Paulus W. Lisanby S. H.. (2012). Fundamentals of transcranial electric and magnetic stimulation dose: definition, selection and reporting practices. Brain Stimul. 5, 435–453. 10.1016/j.brs.2011.10.001 22305345 PMC3346863

[B35] Priori A. Hallett M. Rothwell J. C. (2009). Repetitive transcranial magnetic stimulation or transcranial direct current stimulation? Brain Stimul. 2, 241–245. 10.1016/j.brs.2009.02.004 20633424

[B36] Rees C. S. Maclaine E. (2015). A systematic review of videoconference-delivered psychological treatment for anxiety disorders. Aust. Psychol. 50, 259–264. 10.1111/ap.12122

[B37] Reithler J. Peters J. C. Sack A. T. (2011). Multimodal transcranial magnetic stimulation: using concurrent neuroimaging to reveal the neural network dynamics of noninvasive brain stimulation. Prog. Neurobiol. 94, 149–165. . 10.1016/j.pneurobio.2011.04.004 21527312

[B38] Riper H. Andersson G. Christensen H. Cuijpers P. Lange A. Eysenbach G. (2010). Theme issue on e-mental health: a growing field in internet research. J. Med. Internet Res. 12:e74. 10.2196/jmir.1713 21169177 PMC3057318

[B39] Sauvaget A. Poulet E. Mantovani A. Bulteau S. Damier P. Moutaud B.. (2018). The psychiatric neuromodulation unit: implementation and management. J. ECT 34, 211–219. 10.1097/YCT.0000000000000513 29944606

[B40] Talevi D. Socci V. Carai M. Carnaghi G. Faleri S. Trebbi E.. (2020). Mental health outcomes of the CoViD-19 pandemic. Riv. Psichiatr. 55, 137–144. 10.1708/3382.33569 32489190

[B41] Turgoose D. Ashwick R. Murphy D. (2018). Systematic review of lessons learned from delivering tele-therapy to veterans with post-traumatic stress disorder. J. Telemed. Telecare 24, 575–585. 10.1177/1357633X17730443 28958211

[B42] Vanneste S. Plazier M. Ost J. van der Loo E. Van de Heyning P. De Ridder D. (2010). Bilateral dorsolateral prefrontal cortex modulation for tinnitus by transcranial direct current stimulation: a preliminary clinical study. Exp. Brain Res. 202, 779–785. 10.1007/s00221-010-2183-9 20186404

[B43] Wexler A. (2018). Who uses direct-to-consumer brain stimulation products and why? a study of home users of tDCS devices. J. Cogn. Enhancement 2, 114–134. 10.1007/s41465-017-0062-z

[B44] Woods A. J. Bryant V. Sacchetti D. Gervits F. Hamilton R. (2015). Effects of electrode drift in transcranial direct current stimulation. Brain Stimul. 8, 515–519. 10.1016/j.brs.2014.12.007 25583653 PMC4461479

[B45] Wootton R. (2001). Telemedicine. BMJ 323, 557–560. 10.1136/bmj.323.7312.557 11546704 PMC1121135

[B46] Zis P. Shafique F. Hadjivassiliou M. Blackburn D. Venneri A. Iliodromiti S.. (2020). Safety, tolerability and nocebo phenomena during transcranial magnetic stimulation: a systematic review and meta-analysis of placebo-controlled clinical trials. Neuromodulation 23, 291–300. 10.1111/ner.12946 30896060

